# Secondary traumatic stress and work ability in death care workers: The moderating role of vicarious posttraumatic growth

**DOI:** 10.1371/journal.pone.0289180

**Published:** 2023-07-27

**Authors:** Annalisa Grandi, Marco Rizzo, Lara Colombo

**Affiliations:** Department of Psychology, University of Turin, Turin, Italy; Taipei Medical University, TAIWAN

## Abstract

Death care work consists of dealing with traumatic events frequently, if not daily. This type of exposure is considered characteristic of the profession and can lead to significant negative consequences such as secondary traumatic stress. However, sometimes positive changes can occur as a result of experiencing trauma, which is referred to as vicarious posttraumatic growth. The aim of the present study is to investigate the role of vicarious posttraumatic growth (VPTG) in the relationship between secondary traumatic stress (STS) and work ability (WA) in a sample of 231 death care workers in northern Italy. Regression analysis with interaction was performed using PROCESS. The results showed a negative association between STS and WA and a positive association between VPTG and WA. The interaction between STS and VPTG was also statistically significant. The moderating role of VPTG was partially confirmed by the analysis: at low and moderate VPTG levels, the conditional effect was negative and statistically significant, while at high VPTG, STS exposure had no significant and negative effect on WA, as if VPTG had some kind of protective role against STS. These results provide new insights into the role of VPTG in work environments with daily trauma exposure, such as death care.

## Introduction

Death is a fundamental and inevitable aspect of life, yet dealing with it is a difficult task for many. Dealing with death means confronting our finitude as human beings, the uncertainty of how much time we have left, and the awareness of what will happen to the body we have inhabited when we have left it. Professionals in the field of death care are those who, despite the difficulty of the task, take charge of the entire process of caring for the body–from the moment of death to burial/ash scattering–and relieve the bereaved of some of the burden of loss; in this sense, they could be placed under the category of the helping professions.

Death care encompasses several services, including cremation services, funeral services (such as funeral directors, pallbearers), mortuary services (and forensic work) and cemetery services. Workers in all these areas are constantly exposed to the sight of coffins and/or corpses (in various states of decomposition) and the suffering of the bereaved due to the specific nature of their work [1–7]. Overexposure to trauma and narratives of traumatic experiences can lead to secondary traumatic stress in workers. This type of exposure is considered a serious occupational risk, as trauma in the work context can lead to significant negative effects on psychophysical health, such as anxiety and depression [8]. Trauma work can also significantly affect workers’ job performance [8] and reduce workers’ perceptions of their ability to meet the mental and physical demands of the job [6]. It is important to note that individuals exposed to traumatizing situations at work can also experience positive outcomes. Vicarious posttraumatic growth is possible when the worker is able to find new meanings and develop personal growth after experiencing trauma.

The aim of this study is to examine the relationship between occupational trauma exposure, the capacity of death care workers to overcome traumatic events, and their perceptions of work ability in a currently under-researched work context, such as death care. The theoretical framework used is based on the Job Demands-Resources Theory, JD-R [9] and the Conservation of Resources Theory, COR [10–12]. The first model assumes that a balance between job demands and job and personal resources can reduce negative consequences (not only from a work perspective, such as disengagement, but also in terms of psychophysical health, such as exhaustion) and can have positive effects, e.g. in terms of employees’ health, commitment and job satisfaction [9]. The importance of resources was widely studied and valued by Hobfoll, who argued that people tend to protect their resources and develop new ones, also because they can be useful protective factors during stressful events [12]. In this framework, it is also claimed that resources can mitigate the relationship between demands (threats) and negative outcomes, which is consistent with the assumptions of JD-R theory [13]. According to the JD-R and the theoretical framework of COR, exposure to trauma is a feature of death care and can therefore be considered a job demand. Vicarious posttraumatic growth is the result of a process of living through trauma that leads to the acquisition of a new and deeper view of the world. In this sense, it can be considered a very important resource in the death care, which can help to balance the job demands. Finally, workers’ perception of their own ability to work is another resource that can be undermined by exposure to trauma at work, but at the same time can be enhanced if other resources such as vicarious posttraumatic growth are present.

### Secondary traumatic stress

The literature on trauma and its effects–both short- and long-term–is extensive, but indirect exposure to trauma has only recently been studied. The term secondary traumatic stress (STS) was introduced to describe the negative effects experienced by those in close contact with trauma victims [14]. This category includes workers who are indirectly exposed to trauma because of the specific characteristics of their jobs, such as providing assistance to people who have been physically or psychologically abused, to victims of road, domestic or workplace accidents, and people grieving the loss of a loved one. Emergency workers [15], police officers [16], firefighters [17,18], social workers [19,20], mental health professionals [21,22] and death care workers [2,23], because of their constant exposure to trauma, are among the occupational groups at highest risk for secondary traumatic stress, which is considered a serious occupational risk. The symptomatology to which secondary traumatic stress is attributed is that of post-traumatic stress disorder (PTSD), the main features of which are intrusion–re-experiencing the traumatic material in an unwanted way–avoidance–avoiding emotions or stimuli related to the traumatic event–and arousal, i.e. increased physical excitement/tension [24].

It should be noted that the term secondary traumatic stress is used interchangeably with the term of vicarious traumatization [25]. Although they share some similar features, secondary traumatic stress refers more to "socio-emotional symptoms" [26] and it is associated with PTSD symptoms [27], while vicarious traumatization is considered more of a change in mental patterns due to empathic work with trauma victims [28].

Several studies have shown that STS is related to negative psychophysical outcomes [29], such as anxiety, and depression [8]. Also, some sociodemographic characteristics seem to be related to STS, such as female gender, older age, and seniority [29]. Factors that play a protective role include social support, affective commitment and role clarity, and personal resources such as mindfulness and resilience [29]. Non-functional coping strategies for dealing with STS include instead denial, alcohol and tobacco use, and negative humor [29].

In one of the few studies of death care work STS scores were found to be higher in workers who had less frequent exposure to corpses [2]; STS scores were also higher in workers involved with bereaved families, along with higher scores for secondary traumatic self-efficacy and perceived gratitude, suggesting that these workers may have developed personal resources that can protect them from the negative effects of secondary trauma [2]. Another study of a sample of cemetery workers found that those workers most exposed to traumatic events at work (gravediggers and front office workers) had higher STS scores [23].

### Work ability

The interest in work ability (WA) stems from occupational health research to “predict” work capacity in the aging population. One of the most commonly used definitions of the construct is that of Tuomi and colleagues, namely “How good is the worker at present, in the near future, and how able is he or she to do his or her work with respect to work demands, health, and mental resources?” ([30], p.67). In over 30 years of research on this construct, it has been widely demonstrated that WA is able to predict important aspects of occupational health, such as early retirement [31], disability, and mortality [32]. Recently, efforts have been made to promote WA to improve the quality of life of aging workers. Indeed, a good level of WA is associated with good psychophysical functioning in workers upon retirement [33]. It is important to note that work ability is not a personal characteristic of the worker, but is given by the interaction between job demands and the worker’s personal resources [31]. Research on work ability has focused on various occupational groups, such as teachers [34–36], administrative employees [37], healthcare professionals [38,39], construction workers [40], bus drivers [41], and petrochemical industry employees [42]. Although work in the death care sector is both physically and emotionally demanding, only one study has been conducted. Cotrim and colleagues [6] investigated the psychosocial factors influencing WA in a sample of Portuguese cemetery workers. Compared to other occupational groups in the same country, such as municipal employees, WA scores were lower. The study also showed that burnout, temporary impairment, job satisfaction, and quality of leadership were predictors of WA along with age and general health. In general, few studies have focused on workers exposed to different types of trauma. Results showed lower scores for WA and PTSD symptoms compared to unexposed workers [43–45] and also a negative association between WA and fatigue, workload, and frustration [46].

### Vicarious posttraumatic growth

The term “posttraumatic growth” (PTG) appeared for the first time in the mid-1990s in two works by Tedeschi and Calhoun [47,48]. Posttraumatic growth refers to the positive changes an individual experiences after an event *lived as* traumatic [49,50]. The emphasis on individual perception is a characteristic aspect of PTG. According to the definitions of the psychiatric classification systems, “trauma” is associated with life-threatening events and PTSD symptoms (see [51–53]), that is more focused on objective characteristics. Calhoun and Tedeschi [50], on the other hand, also consider the subjective component and define trauma as “a highly stressful and challenging *life-altering event*” for a particular person ([50] p.8), thus contributing to a broader definition. This extended view is more appropriate as the definition of a traumatic experience can change over time and vary according to culture [50]. Posttraumatic growth can thus occur after a trauma–a crisis or very stressful event–that causes individuals to break their mental patterns, their known world, and create new patterns. The phenomenon consists of five areas: 1) a greater appreciation of life and a changed sense of priorities, 2) warmer relationships, 3) a greater sense of personal strength, 4) recognising new possibilities or paths for one’s life, and 5) spiritual development (for those who are not religious, greater existential questions) [54]. The authors proposed an instrument, the Posttraumatic Growth Inventory (PTGI), to measure and quantify this type of personal growth [48], and more recently developed a short form (PTGI-SF) [55]. To date, this instrument, which has been translated and validated in many languages, is the most widely used to assess posttraumatic growth [50].

While many studies on PTG have been conducted on the general population in the context of the experience of various traumas, such as vehicle accidents survivors [56,57], cancer patients [58,59], bereaved parents [60], disaster survivors [61], refugees [62], prisoners of war [63,64], this phenomenon has also been studied in workers for some years. Certain work contexts are indeed characterized by a strong and frequent–sometimes daily–exposure to traumatic events, such as healthcare, emergency services, social work, death care or the police sector. When the experience of posttraumatic growth is associated with exposure to direct and indirect trauma, it is referred to as vicarious posttraumatic growth (VPTG). Professionals in the above fields work with people who have suffered trauma and are simultaneously affected by the same traumatic events [50]. As emerged from the systematic review by Manning-Jones and colleagues, some features of PTG are recognizable in VPTG, such as changes in life priorities and values, spiritual growth, increased personal growth and improved social relationships. Although VPTG falls under PTG–understood as an umbrella term–it should be noted that it has some distinctive features. The domain into which these unique changes fall is professional identity. In contexts where vicarious posttraumatic growth occurs, significant associations have been observed with higher job value [65], improved occupational skills [66] and, generally, a greater sense of professional competence [67]. Some factors that facilitate VPTG have also been identified. Empathy in the relationship with traumatized clients, for example, appears to help professionals feel the traumatic experience as if it were their own, which facilitates VPTG [67,68]. Positive affect and optimism also promote VPTG. An optimistic and positive outlook on life may more readily lead to recognition of positive outcomes after a vicarious trauma experience, as demonstrated in Linley and Joseph’s study of a sample of funeral directors [69]. Interestingly, in the same study, the authors found that negative affect was also closely associated with VPTG, confirming that it is necessary *to go through* the traumatic experience in order to develop VPTG [69]. Another important factor is self-care, understood as coping strategies to maintain psychophysical wellbeing. Several studies have shown a positive association between VPTG and self-care activities such as exercise, healthy eating habits, hobbies and spiritual practices [67]. Regarding factors affecting the interpersonal domain, social support seems to promote coping and adaptation after trauma and reduce isolation and feelings of loneliness [67]. A final factor that may promote VPTG is time. It seems that the distress experienced by professionals working with trauma decreases over time, in favour of the development of greater personal growth, as if time allows the traumatic event to be integrated and new meanings to be found, thus favouring VPTG [67].

While VPTG has been studied in the context of helping professions, such as healthcare and emergency workers, there are currently few studies conducted in the death care work. The study by Linley and Joseph [69] found that positive changes following contact with death in funeral directors were associated with positive attitudes towards life and social support. Another study looking at funeral work found a higher quality of family relationships, greater understanding of clients’ grief, an approach to death as a natural and inevitable event, and a greater appreciation of life [70]. The positive impact of dealing with death and trauma at work was also found in military mortuary workers, who reported personal growth and improved coping skills as positive outcomes of mortuary experience [71].

**Fig 1:**
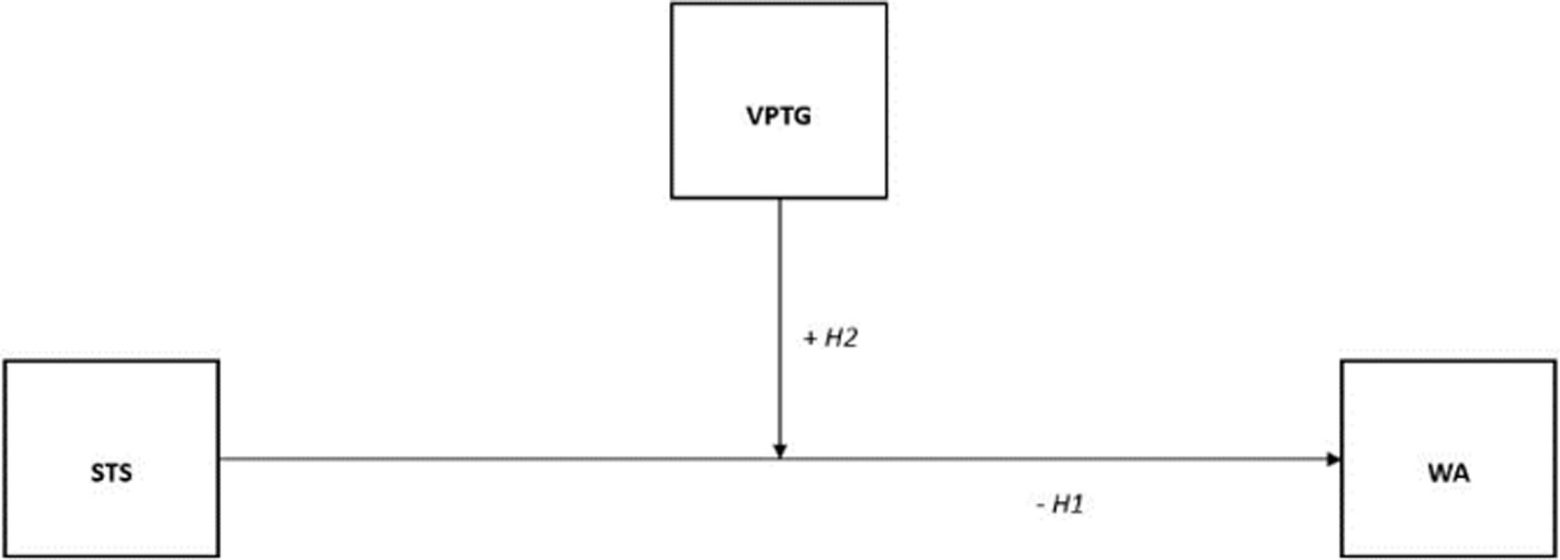
Conceptual model. *Note*. STS = Secondary Traumatic Stress; VPTG = Vicarious Posttraumatic Growth; WA = Work Ability.

Given the results found in the literature, it is of particular interest to delve deeper into the relationship between trauma exposure outcomes, work ability and vicarious posttraumatic growth in a neglected field such as the death care sector. According to the literature discussed so far and the theoretical framework of Job Demands-Resources Theory, JD-R [9] and Conservation of Resources Theory, COR [10–12], our model (see Fig 1) supports the following hypotheses:

*Hypothesis 1 (H1)*: *Secondary traumatic stress (STS) is negatively associated with work ability (WA)*.
*Hypothesis 2 (H2)*: *Vicarious posttraumatic growth (VPTG) has a moderating role in the relationship between secondary traumatic stress (STS) and work ability (WA)*.

## Method

### Participants and procedure

The present study was conducted in accordance with the guidelines of the Declaration of Helsinki (and subsequent revisions) and the ethical requirements of Italian legislation and approved by the Bioethics Committee of the University of Turin (protocol code no. 0598340).

The professional reference sector is complex in terms of organization and accessibility compared to other sectors, therefore a convenience sampling method was chosen and the sample size was not estimated in advance. The funeral agencies of the provincial capital and some neighboring municipalities, the mortuaries of the main provincial hospitals and the municipal mortuary, the provincial offices of the cemeteries and the provincial crematoria were contacted.

An ad hoc questionnaire was prepared and distributed in hard copy to participants at times and dates agreed with employers/managers who agreed to participate in the study. The questionnaire was accompanied by a sheet explaining the aims of the study and how the data would be processed (in accordance with EU Regulation 2016/679). To participate in the study, workers had to read and sign the consent form. The researcher attended the meetings and presented the research project and the objectives of the survey to clarify any doubts the participants might have. The response rate was 90% of the distributed questionnaires. The questionnaire did not provide for the collection of personal data and participants did not receive any compensation; in addition, the consent form signed by the participants was collected separately from the questionnaire so that it was not possible to trace it back to the individual participant.

A total of 259 participants completed the questionnaire. Five participants were excluded because they had not completed at least one of the scales under study. Eighteen participants were excluded because they could not answer the items on secondary traumatic stress because they did not interact with other people during their work. A multivariate test for outliers using the Mahalanobis distance also revealed five participants as outliers, who were then excluded from the analyses.

The final sample to test our hypotheses included 231 participants (70.1% males) aged 20 to 74 years (M = 45.3, SD = 12.2; one case was missing for this variable). The socio-demographic characteristics of the respondents are shown in Table 1. In terms of the profession reported by the participants, the majority worked in funeral services (132; 57.1%), followed by mortuary services (57; 24.0%), cremation services (37; 16.0%), and cemetery services (13; 5.1%). Since six participants worked in more than one service, the sum of these four areas of work exceeds the total number of the sample (cf. Table 1).

**Table 1:** Characteristics of the sample.

	*N*	*%*
**Sex**		
Female	69	29.9
Male	162	70.1
**Marital status** ^**a**^		
Single	85	36.8
Married/Cohabiting	112	48.5
Separated/Divorced or Widowed	33	14.3
**Children** ^**b**^		
Yes	131	56.7
No	96	41.6
**Educational level** ^**c**^		
Middle school diploma	93	40.3
High school diploma	109	47.2
Associate degree or higher	28	12.1
**Professional sector**		
Funeral Services^d^	132	57.1
Cremation Services^d^	37	16.0
Mortuary Services^d^	57	24.7
Cemetery Services^d^	13	5.1

*Note*. N = 231.

^a^ 1 missing value

^b^ 4 missing values

^c^ 1 missing value.

^d^ 3 participants are both in funeral and cemetery services; 1 participant is both in mortuary and cemetery services; 2 participants are in both funeral, mortuary, and cemetery services.

### Measures

The measurement scales used in the study are all validated instruments with good consistency and reliability in the literature. The STS and WA have been used in previous research on death care [2,6,23]. The PTGI was selected because it has been used in samples of helping professions, such as emergency workers [72], that share similar characteristics (e.g. exposure to death and contact with the bereaved).

*Secondary traumatic stress* was measured with the 17 items of the Secondary Traumatic Stress Scale (STSS) developed by Bride and colleagues [27]. The instrument is composed of three subscales, intrusion, avoidance and arousal, but for the purposes of this study only the total score was used. Responses were on a 5-point Likert scale (0 = never; 4 = very often); an example item is “I avoided people, places, or things that reminded me of my work with clients”. The total score of the original scale ranges from 17 to 85. A low or no STS is indicated by a score ≤ 28, a mild level by scores between 28 and 37, a moderate level by scores between 38 and 43, a high level of STS by scores between 44 and 48, and a severe STS by scores ≥ 49 [73]. In the original validation study, the Cronbach’s α was .93, while in this study was .90.

*Work ability* was measured using the Work Ability Index-2 (WAI2) [74], a 2-item scale that asks workers to rate their perceived current work ability in relation to the physical and mental demands of their job on a 5-point Likert scale (0 = very poor; 4 = very good); the total score ranges from 0 to 8.

*Vicarious posttraumatic growth* was measured with the 10 items of the Posttraumatic Growth Inventory-Short Form [55,75] on a 6-point Likert scale (0 = not at all; 5 = very much); the total score ranges from 0 to 50; an example item is “I changed my priorities about what is important in life”. As there is currently no specific measure to assess VPTG, most studies use the same measure developed for direct trauma survivors [67]. When using the scale, reference is not made to personal specific trauma, but to traumatic exposure related to the person’s occupation. The Cronbach’s α was .89 in the original validation study, while in this study was .92.

### Data analysis

Statistical analyses were performed using Statistical Package for the Social Sciences (SPSS 28.0), and a regression model with moderation was tested using Hayes PROCESS (version 4.1, model 1). In a preliminary phase, multiple imputation (MI) was performed after we determined that the data were not missing completely at random. The percentage of missing values for each scale studied ranged from 0.4% to 0.8%. To test our hypotheses, secondary traumatic stress (STS) was the independent variable, vicarious posttraumatic growth (VPTG) was the moderating variable and work ability (WA) was the dependent variable. Having children or not, job tenure, and exposure to bereaved clients (recoded as a dummy according to the median value of the ordinal variable) were used as control variables in the model, as they had been identified in previous studies [76,77] as possible confounders of the relationships under study.

To assess power for moderation analysis with a sample size of 231 and an alpha of 0.05, a post-hoc power analysis was performed using Gpower 3 software [78], with linear multiple regression, fixed model and R^2^ deviation from zero. As recommended, a power of at least 0.80 is acceptable for social science [79].

Frequencies, means, and standard deviations were calculated to summarize the variables included in this study. Pearson correlation (r) was used to test the relationship between variables and results were interpreted according to Cohen’s conventions [80]. Standardized coefficients (ß) and 95% confidence interval (CI) were calculated. The reliability of each scale was determined using the Cronbach’s alpha coefficient with at least three items per scale.

The Johnson-Neyman technique was used to test for statistically significant interactions in the relationship between secondary traumatic stress and work ability. This technique was used to identify areas of significance in the moderator variable (vicarious posttraumatic growth).

## Results

### Preliminary results

Before testing the hypotheses under investigation, some preliminary analyses were performed. The normality assumptions of the regression model were tested. A graphical inspection of the residuals using the Q-Q plot and a scatterplot of the residuals to test homoscedasticity showed no relevant violations of the assumptions. In addition, the Durbin-Watson score (2.09) confirmed the independence of the residuals of the multiple regression models. A test for multicollinearity was performed and the VIF values were all below the cut-off of 5. To test for common method bias, Harman’s single factor test method was used in an exploratory factor analysis. One factor, which included all variables examined, confirmed that the first factor extracted (eigenvalue > 1.0) explained 25.3% of the total variance, less than 40%, which could indicate method bias [81].

The bivariate correlation and descriptive statistics are shown in Table 2.

**Table 2:** Mean, standard deviation, and bivariate correlations between scale study variables.

	*M*	*SD*	1	2	3	4	5	6
1. STS	0.70	0.59	—	-0.06	-0.36***	0.09	0.08	-0.11
2. VPTG	2.80	1.24		—	0.19**	0.17**	0.02	-0.19**
3. WA	6.23	1.39			—	0.01	-0.05	0.06
4. Job tenure	12.38	10.84				—	—	—
5. Children	—	—					—	—
6. Exposure to bereaved clients	—	—						—

*Note*. N = 231. STS = Secondary Traumatic Stress; VPTG = Vicarious Posttraumatic Growth; WA = Work Ability.

***p < 0.001

**p < 0.01

*p < 0.05.

STS was not correlated with VPTG, while a negative correlation was found between STS and WA, VPTG was positively correlated with WA. Among the control variables, a significant negative correlation was found only between VPTG and exposure to bereaved clients (0 = exposure).

**Fig 2:**
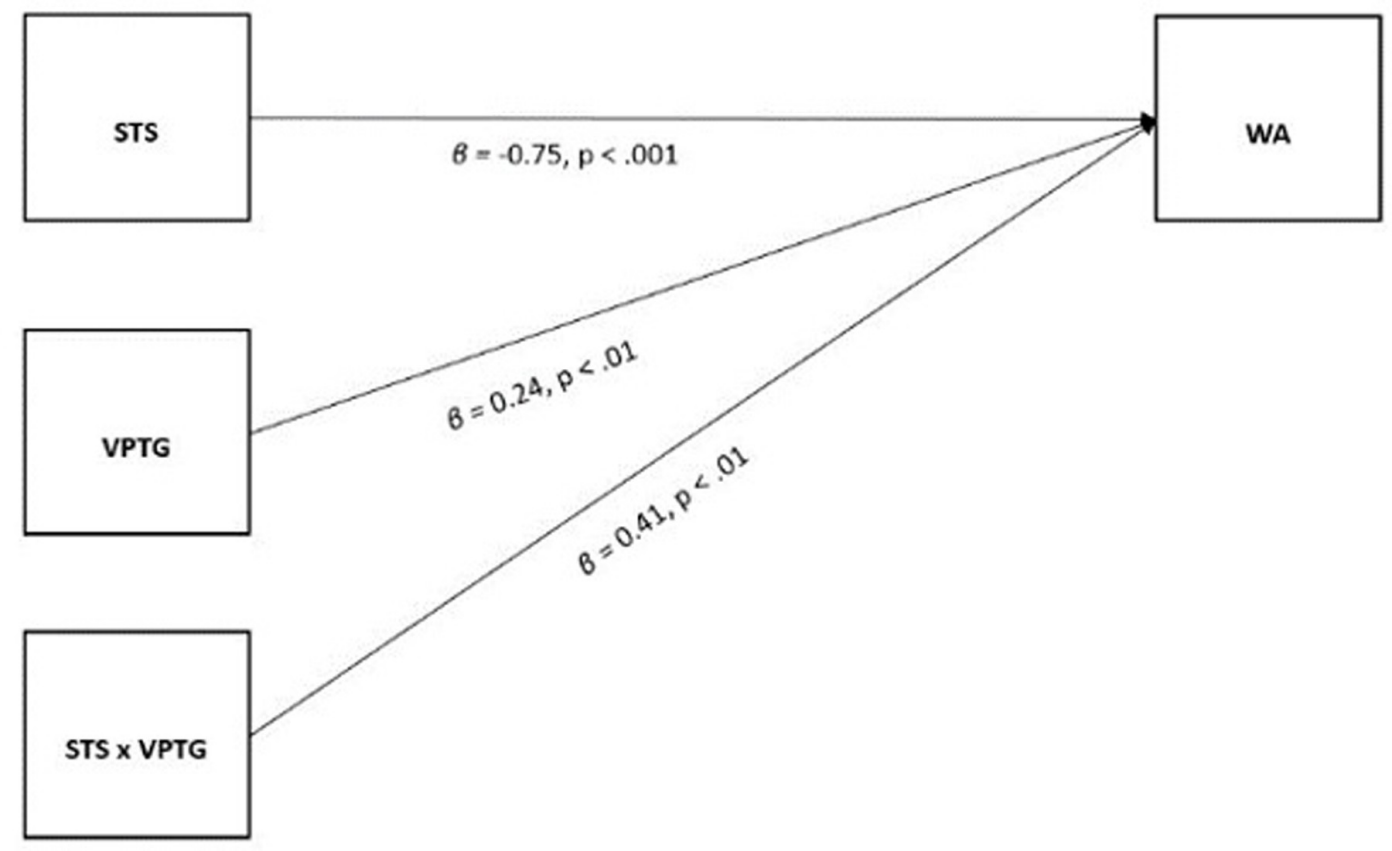
Results of the regression model with interaction effect. *Note*. STS = Secondary Traumatic Stress; VPTG = Vicarious Posttraumatic Growth; WA = Work Ability.

Regarding the results of the moderation model (see Fig 2), the hypotheses were partially confirmed.

The direct association between STS and WA was negative and statistically significant (B = -.75, S.E. = .15, 95% C.I. (-1.03, -.46), p < .001), confirming H1. The association between VPTG and WA was positive and statistically significant (B = .24., S.E. = .07, 95% C.I. (.10, .39), p <. 01). The interaction between STS and VPTG was positive and statistically significant (B = .41., S.E. = .12, 95% C.I. (.17, .65), p < .01). Together, the variables accounted for approximately 20% of the variance, R2 = .20, F (6,219) = 9.3, p < .001. A non-significant association emerged between the control variables and WA.

**Fig 3:**
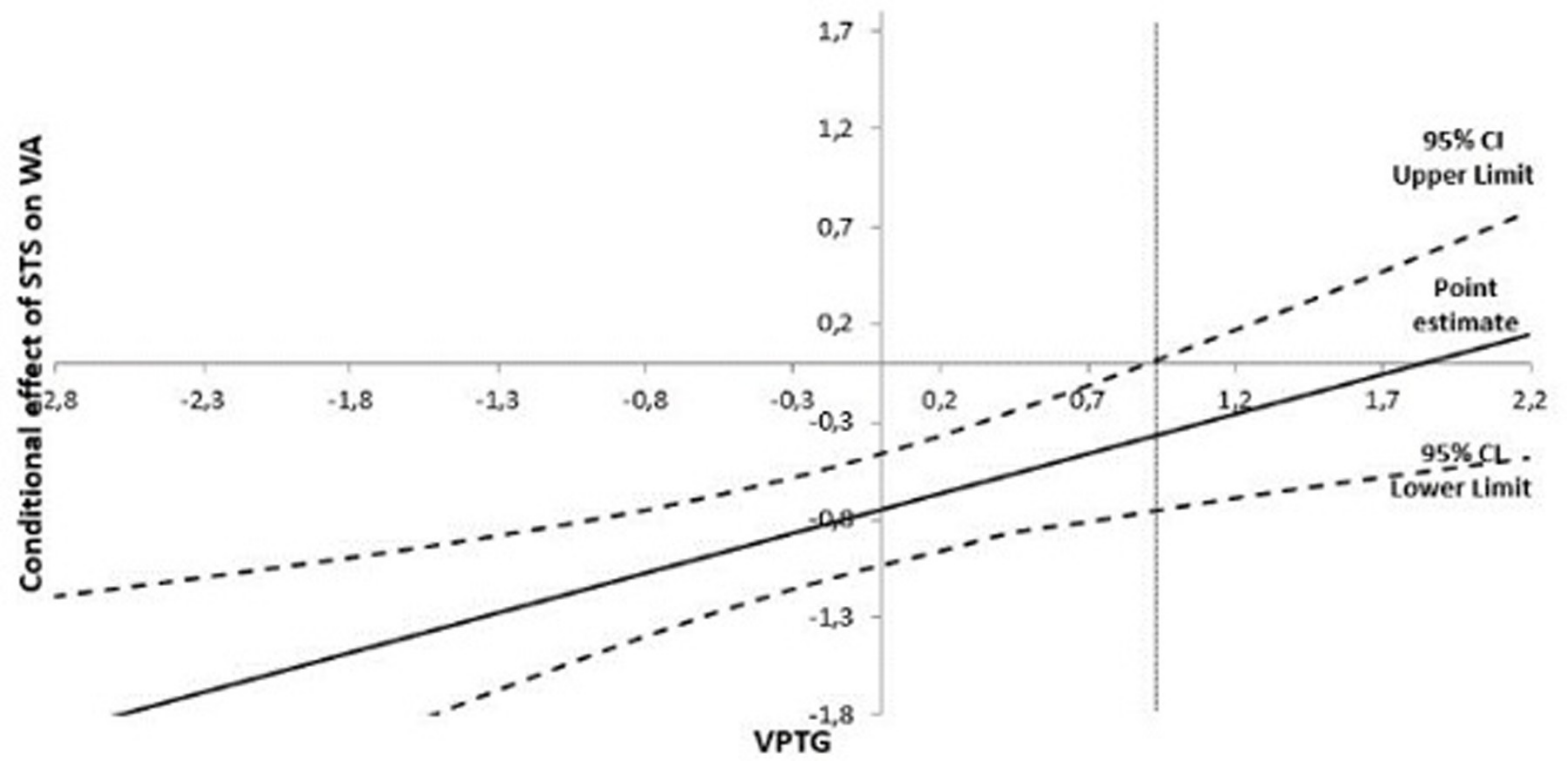
Results of Johnson-Neyman technique.

Regarding the conditional effect of the interaction term, the Johnson-Neyman (see Fig 3), the technique showed that the effect of STS on WA was significant for values of VPTG below 0.90, which partially confirms H2. This implies that the effect of STS on WA is strongly negative when the moderator (VPTG) is low. When the moderator (VPTG) is high, the effect of STS on WA towards 0.

## Discussion

The aim of the present study was to better understand the role of vicarious posttraumatic growth in workers who are continuously exposed to trauma at work. Given the lack of studies in the literature, the results obtained are particularly interesting and contribute to new knowledge about this occupational field.

The first hypothesis of the study (H1) states a negative relationship between secondary traumatic stress and work ability. As we have seen, secondary traumatic stress corresponds to the symptomatological framework of PTSD and is experienced by people who live traumatic situations indirectly, for example through their work. Higher levels of activation, intrusive thoughts of traumatic memories and avoidance behaviours towards emotions or stimuli that remind people of traumatic events are symptoms that can make workers more vulnerable and affect their ability to work in the long term. This hypothesis was confirmed by the analyses, and although the association between STS and WA has not been examined in previous studies in the field of death care work, this finding is consistent with other studies in other professions that deal with trauma. For example, in the study by Bock and colleagues [8,82], nurses who reported more severe STS symptoms reported higher levels of depression, anxiety and work stress, and lower work ability; and in another study of police officers working in child protection, work ability was a good predictor of secondary trauma [83].

Regarding the second hypothesis (H2), we have argued that vicarious posttraumatic growth plays a moderating role in the relationship between STS and WA. VPTG describes the set of positive changes that can occur as a result of experiencing traumatic events directly and indirectly. A distinguishing feature of the construct is the necessity of *living through* the traumatic experience: it is not possible to avoid it. With this in mind, it was necessary to consider symptoms related to working with trauma in the model. The results of the analysis partially confirmed the second hypothesis (H2). VPTG indeed seems to have a significant moderator role in the relationship between STS and WA when VPTG scores are low or moderate; its role as a moderator loses statistical significance when VPTG scores are higher. This result is interesting because it suggests the protective role of VPTG against STS. It underscores that stress related to traumatic experience loses significance when individuals have experienced growth. This finding is consistent with previous research on workplace trauma, which found that VPTG moderated the effects of STS in therapists who worked with trauma survivors [84]. The study highlights the stress-buffering effect of VPTG and suggests that higher VPTG may have helped therapists reinterpret challenging and threatening aspects of their work, leading to a lower experience of secondary traumatic stress [84]. Moreover, this result seems to be consistent with the definition of the VPTG construct; the kind of growth that VPTG leads to indeed includes a greater appreciation of life, a changed sense of priorities, and warmer relationships; all of these new achievements can contribute to living difficult work environments, such as those of trauma-work, in a more balanced way and consequently experiencing lower levels of stress. A greater sense of personal strength, typical of post-traumatic vicarious growth, may be another element that improves workers’ perceptions of their ability to do their work in terms of available physical energy and required mental energies.

These results are also in line with the proposed theoretical framework. We have seen that occupational exposure to trauma is one of the typical work demands in death care. This psychosocial risk factor may lead to an increase in perceived work ability where there is growth of the individual. This confirms the protective role of resources in relation to job demands as supported by the JD-R model, as well as the gain spiral created by acquiring new resource, as postulated by the COR model.

These findings highlight the importance of supporting personal growth processes in a work environment where individuals are constantly exposed to traumatic events.

## Conclusions

Death care is a professional sector that has received little research attention, making it essential to better understand the protective factors that can maintain employee wellbeing.

The findings of the present study provide new insights into the role of vicarious posttraumatic growth in work environments with daily trauma exposure and suggest that workers who develop these positive changes may improve their wellbeing and their work ability.

These results are particularly interesting in light of recent events related to the COVID-19 situation, where workload and trauma exposure have increased exponentially, severely scarring all workers involved in the frontline management of the emergency, including death care workers [85,86]. Identifying the determinants of wellbeing in the workplace may allow targeted interventions to be designed, even at job induction, to provide workers with useful resources to cope with the negative consequences of the traumatic experience [87,88]. For example, supporting the vicarious posttraumatic growth process and raising awareness of the importance of personal and professional self-care practices may be useful interventions to enhance wellbeing in the workplace.

Although the present study has brought new insights to the field of death care research, it is also important to note its limitations. Firstly, the sample size is not very large and a convenience sample was used. In addition, due to of the cross-sectional design, it was not possible to test causal relationships between variables.

Some other information, such as socioeconomic status, health status, access to psychological, social, medical and structural resources, perceived severity of working with trauma survivors and personal trauma history, was not collected; future studies could also include these variables, which could play an interesting role in the proposed model. Furthermore, because the sample was not evenly distributed across professional groups, it was not possible to examine differences in the association between the model variables in the death care professional groups. Future studies could examine these differences. Another limitation is that the instrument used to measure secondary traumatic stress, namely the STSS, is based on the DSM-4 PTSD symptomatology. However, it is also important to note, that the literature argues that the DSM-4 symptoms can still approximately measure the DSM-5 diagnosis [89].

Finally, in the future it would be interesting to use samples from different countries to also investigate cross-cultural differences and to think about longitudinal models to also explore the dimensions of causality.

## Supporting information

S1 File Questionnaire. (PDF)

S1 Dataset Dataset. (SAV)
