# Supplementary material for: Secondary traumatic stress and work ability in death care workers: The moderating role of vicarious posttraumatic growth
Source: PLoS One. 2023 Jul 27;18(7):e0289180. doi: 10.1371/journal.pone.0289180 (PMC10374133; doi:10.1371/journal.pone.0289180)
Supplement: S1 File — (PDF) [file pone.0289180.s001.pdf]

At the beginning we ask you for some information about your socio-anagraphic situation.

A1. Age: (years) ……………………………….

A2. Sex: (0) ❒ female (1) ❒ male

A3. Marital status: (1) ❒ single (2) ❒ married/cohabiting
(3) ❒ separated/divorced (4) ❒ widowed

A4. Do you have children? (0) ❒ no (1) ❒ yes

A5. Educational level: (1) ❒ middle high school diploma (2) ❒ high school diploma
(3) ❒ associate degree (4) ❒ higher degree

A6. What is your professional role? (Available more options)

A6.1 ❒ Cemetery services

A6.2 ❒ Mortuary services

A6.3 ❒ Cremation services

A6.4 ❒ Funeral services

A7. How long have you been working in the funeral business? (years)

(if less than one year, indicate zero)

……………………………

A8. When you think about your work, how often do you deal with the familiars of deceased people?

(1) ❒ daily (2) ❒ a few times a week (3) ❒ a few times a month (4) ❒ never

Thinking about your work and your relationship with users, please read each statement, then indicate how frequently the statement was true for you in the <u>past seven (7) days.</u>

		Never	*Rarely*	*Occasionally*	*Often*	*Very often*
**B1.**		0	1	2	3	4
B1.1	I felt emotionally numb	❒	❒	❒	❒	❒
B1.2	My heart started pounding when I thought about my work with clients	❒	❒	❒	❒	❒
B1.3	It seemed as if I was reliving the trauma(s) experienced by my client(s)	❒	❒	❒	❒	❒
B1.4	I had trouble sleeping	❒	❒	❒	❒	❒
B1.5	I felt discouraged about the future	❒	❒	❒	❒	❒
B1.6	Reminders of my work with clients upset me	❒	❒	❒	❒	❒
B1.7	I had little interest in being around others	❒	❒	❒	❒	❒
B1.8	I felt jumpy	❒	❒	❒	❒	❒
B1.9	I was less active than usual	❒	❒	❒	❒	❒
B1.10	I thought about my work with clients when I didn't in-tend to	❒	❒	❒	❒	❒
B1.11	I had trouble concentrating	❒	❒	❒	❒	❒
B1.12	I avoided people, places, or things that reminded me of my work with clients	❒	❒	❒	❒	❒
B1.13	I had disturbing dreams about my work with clients	❒	❒	❒	❒	❒
B1.14	I wanted to avoid working with some clients	❒	❒	❒	❒	❒
B1.15	I was easily annoyed	❒	❒	❒	❒	❒
B1.16	I expected something bad to happen	❒	❒	❒	❒	❒
B1.17	I noticed gaps in my memory about client sessions	❒	❒	❒	❒	❒

How do you assess your current professional skills in relation to the...

B2.		*Very poor* 0	*Mediocre* 1	*Medium* 2	*Good* 3	*Very Good* 4
B2.1	Physical energies required by his/her work	❐	❐	❐	❐	❐
B2.2	Mental energies required by his/her work	❐	❐	❐	❐	❐

Thinking about your experiences working in the funeral business over the years, please indicate the extent to which you have noticed the following changes in your life.

B3.		*Not at all* 0	1	2	3	4	*Very much* 5
B3.1	I changed my priorities about what is important in life	❐	❐	❐	❐	❐	❐
B3.2	I have a greater appreciation for the value of my own life	❐	❐	❐	❐	❐	❐
B3.3	I am able to do better things with my life	❐	❐	❐	❐	❐	❐
B3.4	I have a better understanding of spiritual matters	❐	❐	❐	❐	❐	❐
B3.5	I have a greater sense of closeness with others	❐	❐	❐	❐	❐	❐
B3.6	I established a new path for my life	❐	❐	❐	❐	❐	❐
B3.7	I know better that I can handle difficulties	❐	❐	❐	❐	❐	❐
B3.8	I have a stronger religious faith	❐	❐	❐	❐	❐	❐
B3.9	I discovered that I’m stronger than I thought I was	❐	❐	❐	❐	❐	❐
B3.10	I learned a great deal about how wonderful people are	❐	❐	❐	❐	❐	❐

Thank you for taking the time to complete the questionnaire and thus contributing to the successful completion of the present study!
